# Liver proteome response of pre-harvest Atlantic salmon following exposure to elevated temperature

**DOI:** 10.1186/s12864-018-4517-0

**Published:** 2018-02-12

**Authors:** Waldo G. Nuez-Ortín, Chris G. Carter, Peter D. Nichols, Ira R. Cooke, Richard Wilson

**Affiliations:** 1 Institute for Marine and Antarctic Studies, University of Tasmania, Private Bag 49, Hobart, TAS 7001 Australia; 2 CSIRO Food Nutrition and Bio-based Products, Oceans & Atmosphere, GPO Box 1538, Hobart, TAS 7001 Australia; 3 Comparative Genomics Centre, James Cook University, Townsville, QLD 4811 Australia; 4 Central Science Laboratory, University of Tasmania, Bag 74, Hobart, TAS 7001 Australia

**Keywords:** Proteomics, Heat stress, Aquaculture, Climate change

## Abstract

**Background:**

Atlantic salmon production in Tasmania (Southern Australia) occurs near the upper limits of the species thermal tolerance. Summer water temperatures can average over 19 **°**C over several weeks and have negative effects on performance and health. Liver tissue exerts important metabolic functions in thermal adaptation. With the aim of identifying mechanisms underlying liver plasticity in response to chronic elevated temperature in Atlantic salmon, label-free shotgun proteomics was used to explore quantitative protein changes after 43 days of exposure to elevated temperature.

**Results:**

A total of 276 proteins were differentially (adjusted *p*-value < 0.05) expressed between the control (15 **°**C) and elevated (21 **°**C) temperature treatments. As identified by Ingenuity Pathway Analysis (IPA), transcription and translation mechanisms, protein degradation via the proteasome, and cytoskeletal components were down-regulated at elevated temperature. In contrast, an up-regulated response was identified for NRF2-mediated oxidative stress, endoplasmic reticulum stress, and amino acid degradation. The proteome response was paralleled by reduced fish condition factor and hepato-somatic index at elevated temperature.

**Conclusions:**

The present study provides new evidence of the interplay among different cellular machineries in a scenario of heat-induced energy deficit and oxidative stress, and refines present understanding of how Atlantic salmon cope with chronic exposure to temperature near the upper limits of thermal tolerance.

**Electronic supplementary material:**

The online version of this article (10.1186/s12864-018-4517-0) contains supplementary material, which is available to authorized users.

## Background

A major impact of climate change on fisheries and aquaculture is the increased seawater temperature she [1]. Aquatic ectotherm species in the temperate areas are particularly vulnerable given the high warming rate of this area [2]. In Tasmania (Southern Australia), seawater Atlantic salmon is produced near the upper limits of thermal tolerance [3]. Post-smolt Atlantic salmon are generally considered to have an optimum temperature range of 13–15 °C for growth and an upper critical range of 22–33 °C. [4], while the sea surface temperature in Tasmania averages over 19 °C for several weeks during the summer period (IMOS-OceanCurrent, unpublished data) and further increases are projected [5]. Sea-caged Atlantic salmon cannot escape the increased surface water temperatures by vertical migration and thus risk long-term exposure to heat stress and physiological challenges that both impair production efficiency and raise welfare issues [6].

Heat stress co-occurs with hypoxia, and although oxygen limited thermal tolerance is of major importance in the fish physiological response [7], the temperature-dependent response occurs independently of water oxygen saturation [8, 9]. Any temperature rise increases the metabolic rate and consequently the maintenance of energy requirements, leading to a state of metabolic remodeling to compensate for increased energy demand [10]. This response is dependent on the species’ thermal tolerance (eurythermals vs. stenothermals) and the exposure regime (acute vs. short-term vs. long-term) [11]. Individual tissues show highly divergent responses to thermal stress that are likely related to their physiological role in the body [11]. The liver is the central hub for the regulation of nutrient metabolism and detoxification [12], and thus constitutes an excellent target to characterize mechanisms of acclimation to chronically elevated temperature. To our knowledge, the molecular plasticity of liver of Atlantic salmon exposed to chronically elevated temperature is currently limited to analysis at the transcriptome level [13]. The most notable findings of this study were reduced protein synthesis and increased xenobiotic metabolism in fish held at 19 **°**C. Shotgun proteomics can provide an unprecedented view of the liver response of this species to chronic heat stress, and offers potential to unravel adaptive physiological mechanisms that can be only postulated by the transcriptome. In addition, biological interpretation of data generated from Atlantic salmon is greatly benefited from increased genomic resources such the recently published reference genome [14], associated protein database improvements (e.g. UniProt), and the continuous improvement of the bioinformatics tools used for gene ontology (GO) enrichment (e.g. Ingenuity Pathway Analysis, DAVID). In relation to previous fish (i.e. non-salmon species) proteomics research, the liver response to thermal stress has been only examined using gel-based approach [15, 16]. Shotgun proteomics can extend the range of quantifiable proteins and provide a more detailed characterization of the affected mechanisms that are common to different species and thermal stresses.

Here we explored the long-term exposure to elevated temperature on the molecular response in liver tissue of pre-harvest Tasmanian Atlantic salmon using label-free shotgun proteomics. We compared two water temperatures, 15 °C and 21 °C, under the same oxygen saturation levels and after 43 days of exposure. Quantitative changes in the protein expression pattern can contribute to the mechanistic understanding of how Atlantic salmon specifically, and fish generally, cope with chronically elevated temperatures. This study expands fundamental and applied information about fish nutrition under limiting environmental conditions previously conducted in Tasmania [17–20]. These findings may be also of value for the aquaculture industry in further development of dietary formulations for Atlantic salmon specific to the summer period.

## Methods

### Growth trial and sampling

The trial was conducted at the Experimental Aquaculture Facility (EAF) of the Institute for Marine and Antarctic Studies, University of Tasmania (Taroona, Tasmania, Australia) in accordance with University of Tasmania Animal Ethics (Investigation A0015208). All female Atlantic salmon post-smolt (average weight ± STD: 961 ± 172 g) from a single cohort were sourced from a commercial hatchery (Huon Aquaculture, Tasmania, Australia), haphazardly allocated amongst 6 × 2500 L circular tanks at an initial stocking density of 19 fish tank^− 1^ and acclimated for 38 days. Each tank had an independent recirculation system equipped with a heat-exchanger, protein skimmer, drum filter, UV filter and biological filter. Seawater was continually supplied and progressively replaced 1.5 times hour^− 1^ with 10% fresh water exchange day^− 1^. Water temperature was maintained at 15 °C during acclimation. Photoperiod was maintained at 12 h light:12 h dark. Water quality parameters (dissolved oxygen, pH, nitrate and nitrite) were recorded daily and maintained within limits for Atlantic salmon [21]; dissolved oxygen was maintained at 101 ± 0.8% and pH at 7.8 ± 0.0. Fish were fed a commercial diet (Optiline 8 mm, Skretting, Tasmania, Australia) in excess four times day^− 1^ with automatic feeders, and uneaten pellets were collected after termination of each meal to calculate daily feed intake. The feed contained 42% crude protein, 29% lipid, and 21.4 MJ kg^− 1^ digestible energy.

In order to examine the response to elevated chronic temperature, fish were exposed to two temperatures, 15 ± 0.0 °C and 21 ± 0.1 °C. Temperature in triplicate tanks was steadily increased (0.5 °C day^− 1^) over 13 days until it reached 21 °C, and then maintained for 43 days until the end of the experiment. At the beginning of the experiment, fish were anaesthetized (Aqui-S® 50 mg L^− 1^) [22] and benchmarked (wet weight, fork length and skin/fin condition). At the end of the experiment fish were euthanized (Aqui-S® 500 mg L^− 1^) and re-measured for wet weight and fork length. No signs of pathogenic infection to skin or gill were found in any of the treatments. Livers were dissected and weighed, immediately frozen in liquid nitrogen, and stored at − 80 °C for proteomic analysis. Growth rate was correlated with condition factor (Pearson’s r = 0.69, *p* < 0.01) and livers of fish with k < 1.2 (11% of fish at the end of the trial) were discarded from proteomic analysis in order to avoid confounding effects of poor fish condition on protein expression. The effect of elevated temperature on growth biometry and liver proteome was therefore assessed on an individual basis in three fish tank^− 1^ (nine treatment^− 1^) randomly selected among those with k > 1.2.

### Liver preparation for proteomic analysis

#### Protein extraction

Livers (~ 60 mg of frontal lobe tissue) from each treatment group (nine treatment^− 1^) were individually homogenized for 5 s in Eppendorf tubes containing lysis buffer (7 M urea, 2 M thiourea, 50 mM pH 8 Tris) and protease inhibitor cocktail (Roche, NSW, Australia) using Tissue-Tearor homogenator (Biospec Products, OK, USA). Each extraction was performed for 18–24 h at 4 °C with overnight rotation. After removal of insoluble material by centrifugation (13,000 rpm, 15 min at 4 °C), an aliquot was precipitated with 100% ethanol (9:1, *v*/v) overnight. Protein pellets were washed twice in 70% ethanol and re-suspended in lysis buffer. Protein concentrations were estimated with Bradford Protein Assay (Bio-Rad, NSW, Australia) using plate reader (Synergy TMHT, BioTek, QLD, Australia) and the volumes were adjusted with lysis buffer to achieve a concentration of 1 μg μL^− 1^ for each extract.

#### Nano-liquid chromatography and tandem mass spectrometry (LTQ-Orbitrap XL)

Protein samples were trypsin-digested using standard procedures [23] and analyzed by nanoLC-MS/MS using an LTQ-Orbitrap XL and Ultimate 3000 RSLCnano HPLC system (ThermoFisher Scientific, MA, USA). Tryptic peptides (~ 1 μg) were loaded onto a 20 mm × 75 μm PepMap 100 trapping column (3 μm C_18_) at 5 μl/min, using 98% water, 2% acetonitrile and 0.05% TFA. Peptides were separated at 0.3 μl/min on a 250 mm × 75 μm PepMap 100 RSLC column (2 μm C_18_) held at 40 °C, using a stepped gradient from 97% mobile phase A (0.1% formic acid in water) to 50% mobile phase B (0.08% formic acid in 80% acetonitrile and 20% water) comprising 3–10% B over 10 min, 10–40% B over 180 min, 40–50% B over 10 min, holding at 95% B for 10 min then re-equilibration in 3% B for 15 min. The LTQ-Orbitrap XL was controlled using Xcalibur 2.1 software in data-dependent mode and MS/MS spectra were acquired as described [23].

#### Database searching and criteria for protein identification

RAW files from the LTQ-Orbitrap were imported into MaxQuant software version 1.5.1.2 for peptide matching to MS/MS spectra and label-free protein quantification using the max LFQ algorithm [24]. MS/MS spectra were searched against the Salmonidae database (http://uniprot.org/taxonomy/8030; 17,795 entries) using the Andromeda search engine. Default settings for protein identification were used, including a maximum of two missed cleavages, mass error tolerances of 20 ppm then 4.5 ppm for initial and main peptide searches, respectively, 0.5 Da tolerance for fragment ions, variable oxidation of methionine and fixed carbamidomethylation of cysteine. The false discovery rates (FDR) for peptide-spectrum matches and protein identification were both set to 0.01. MaxQuant output files of the complete peptide and protein-level mass spectrometry are provided in Additional files 1 and 2, respectively.

#### Calculations and statistical analysis

Standard formulae were used to assess growth biometrical data. Fulton’s condition factor was calculated as k = W / FL^3^, where W is fish wet weight (g) and FL is fork length (cm). Hepato-somatic index was determined as HSI = (LW / W) × 100, where LW is liver weight (g) and W is fish wet weight (g).

Statistical analyses of biometrical data was performed using R software [25]. Individual fish data was analysed using the Generalized Estimating Equations (GEE) model to control the cluster (tank) correlation derived from the sampling of individuals from different tanks within each treatment [26]. Tank and fish nested within tank were considered random variables. The Wald test was used to detect significant differences (*p* < 0.05) between treatments and results were expressed as mean ± standard error (SEM) (*n* = 9).

For statistical analysis of LTQ-Orbitrap mass spectrometry, the “ProteinGroups” output file generated by MaxQuant analysis of liver extracts was analysed in R [25] using the *limma* package [27]. Proteins identified on the basis of a single matching peptide were excluded and only proteins detected in at least six out of nine biological replicates in any one treatment group were considered. The effect of temperature was investigated by fitting a linear model with log2 protein group intensity as the response and including tank and fish nested to tank as explanatory random variables. Prior to model fitting, intensity values were normalized using cyclic loess normalization [28] and the method of empirical array quality weights [29] was used to calculate sample reproducibility and down-weight less reproducible samples. After initial model fitting, empirical Bayes [30] was used to calculate moderated test statistics and Benjamini Hochberg correction was applied to adjust *p*-values for multiple testing. Missing values for all remaining proteins were excluded from the analysis with degrees of freedom adjusted accordingly. To gain further insight into the potential mechanisms of the effects of elevated temperature, differentially (adjusted p-value < 0.05) expressed proteins were selected for Ingenuity Pathway Analysis (IPA, https://qiagenbioinformatics.com/products/ingenuity-pathway-analysis/). The salmonidae genes were first mapped to human orthologues using PANTHER [31], and then gene symbols and the corresponding fold change in protein expression were submitted to IPA for identification of canonical and toxicity pathways and mapping of interaction networks.

## Results

### Biometric indices

Elevated temperature had a significant and negative effect on k and HSI, although this effect was not significantly reflected in final weight (Table 1). Feed intake (measured by tank) was not significantly affected by elevated temperature.

**Table 1:** Biometric indices of pre-harvest Atlantic salmon (selected on the basis of k^1^ > 1.2) held at 15 °C and 21 °C

	15 °C	21 °C	Test (*p*-value, *Wald*^*2*^)
Final wet weight (g)	2367.1 ± 140.8	2064.9, 104.10	0.140, −
Final length (mm)	523.9 ± 7.8	565.0, 13.85	0.756, −
k^1^	1.6 ± 0.06	1.4 ± 0.07	0.028, 4.834
HSI^3^	1.2 ± 0.0	1.0 ± 0.07	0.000, 14.228

Data expressed as mean ± SEM (*n* = 9). Each replicate represented by an individual fish

^1^Condition factor

^2^Wald estimator only reported when *p*-value < 0.05

^3^Hepato-somatic index

Table 1.

### Liver proteomics

**Fig. 1:**
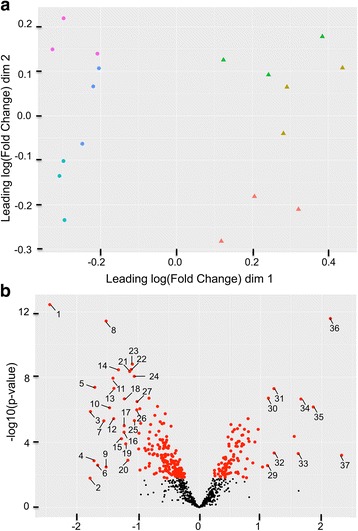
Multidimensional scaling (MDS) (A) and volcano plot (B) of the liver proteome profile of Atlantic salmon held at 15 °C and 21 °C. **a** Data points in the MDS are plotted with symbols (**•**: 15 °C; ▲: 21 °C), representing temperature treatments, and colors, representing tank allocation. **b** Red dots in the volcano plot represent proteins to be significantly different (adjusted *p*-value < 0.05). Numbered dots refer to proteins displaying fold changes > 2 as described in Table 2

**Fig. 2:**
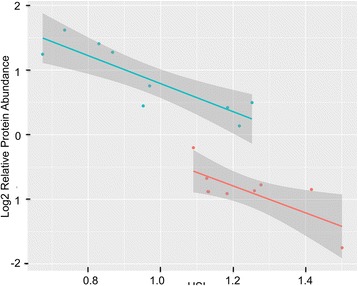
Relative abundance of ferritin (FTL) relative to hepato-somatic index (HSI). Color indicates temperature treatment (Blue: 21 °C; Red: 15 °C). Shaded areas represent 95% confidence intervals. Pearson correlation coefficient across temperature treatments equals to 0.94

A total of 842 proteins were identified on the basis of two or more unique matching peptide sequences and presence in at least six of the nine biological replicates in either treatment group (Additional file 3). Peptide length ranged from seven to 43 residues and averaged 15 residues. Multidimensional scaling (MDS) showed maximum separation between temperature treatments (Figure 1a). On the basis of an adjusted *p*-value < 0.05, comparison of intensity values identified differences in 276 proteins, which are shown on the volcano plot in Fig. 1b. Of these proteins, 89 and 187 proteins were up-regulated and down-regulated in 21 °C livers relative to 15 °C livers, respectively, with fold changes ranging from 1.2 to 5.4. Differentially abundant proteins showing fold changes > 2 are listed in Table 2. One protein, ferritin (FTL), showed a significant (adjusted *p*-value < 0.05) degree of correlation (Pearson’s r = 0.92) with the measured HSI (Fig. 2) in addition to being significantly upregulated at the higher temperature.

**Table 2:** List of differentially abundant proteins with fold changes > 2 in liver of pre-harvest Atlantic salmon held at 15 °C and 21 °C

#	Protein name *(Entry name)*	Fold change 21 °C vs. 15 °C	Unique peptides	adj. *p*-value^1^	Gene name	Human orthologue
1	Acyl-coenzyme A oxidase *(C0H935)*	−5.43	14	< 0.001	ACOX3	ACOX3
2	Leukocyte cell-derived chemotaxin 2 *(B5XCD7)*	−3.44	6	0.050	LECT2	LECT2
3	Betaine-homocysteine methyltransferase *(B5DGE7)*	−3.43	13	< 0.001	bhmt	BHMT
4	5′-nucleotidase (EC 3.1.3.5) *(B5DGD0)*	−3.29	3	0.009	5NT3L	NT5C3A
5	Uricase (EC 1.7.3.3) (Fragment) *(Q3S563)*	−3.26	16	< 0.001		
6	Cytochrome P450 2 M1 *(B5X2R4)*	−3.16	7	0.014	CP2M1	
7	Tubulin folding cofactor B *(B5X4J7)*	−2.95	9	< 0.001	TBCB	TBCB
8	Elongation factor 2 *(C0H9N2)*	−2.87	9	< 0.001	EF2	EEF2
9	Sulfotransferase (EC 2.8.2.-) *(B5X695)*	−2.87	10	0.016	ST2S2	ST2S2
10	Pyruvate kinase (EC 2.7.1.40) *(C0HBL8)*	−2.76	28	< 0.001	KPYK	KPYK
11	High mobility group protein B3 *(C0HBT7)*	−2.66	3	< 0.001	HMGB3	HMGB3
12	Costars family protein ABRACL (ABRA C-terminal-like protein) *(ABRAL)*	−2.63	3	< 0.001		
13	Apolipoprotein A-I (B5XBH3)	−2.63	15	< 0.001	APOA1	APOA1
14	Heat shock cognate 70 kDa protein *(B5DFX7)*	−2.50	9	< 0.001	HSP70	HSPA8
15	Stathmin *(B5X953)*	−2.41	3	0.001	STMN1	STMN1
16	Proliferating cell nuclear antigen *(B9EMQ6)*	−2.34	6	< 0.001	PCNA	PCNA
17	Translationally-controlled tumor like protein *(B5XAC1)*	−2.34	7	< 0.001	TCTP	TPT1
18	Guanidinoacetate N-methyltransferase *(B5DGB5)*	−2.33	5	< 0.001	GAMT	GAMT
19	Lipase *(B5X16)*	−2.30	7	0.002	LICH	LIPA
20	NDRG1 *(B5X292)*	−2.24	3	0.008	NDRG1	NDRG1
21	Proactivator polypeptide *(B5X4D6)*	−2.20	7	< 0.001	SAP	PSAP
22	Peroxisomal trans-2-enoyl-CoA reductase *(B5XAK8)*	−2.16	7	< 0.001	PECR	PECR
23	Elongation factor-1 delta-1 *(B5DGP8)*	−2.13	5	< 0.001		
24	Lupus La protein homolog B *(C0HAU7)*	−2.09	8	< 0.001	LAB	
25	Adenosine kinase a *(B5DGF0)*	−2.08	18	< 0.001		
26	Plasminogen activator inhibitor 1 RNA-binding protein *(B5X326)*	−2.03	4	< 0.001	PAIRB	SERBP1
27	Beta-carotene oxygenase 2 like *(K8DW80)*	−2.02	6	< 0.001	bco2	BCO2
28	Apolipoprotein B (Fragment) *(Q91480)*	2.05	8	0.016		APOB
29	UDP-glucuronosyltransferase (EC 2.4.1.17) *(B5X180)*	2.16	4	0.014	UD2A2	UGT2A1
30	15-hydroxyprostaglandin dehydrogenase *(B9EPG3)*	2.19	11	< 0.001	PGDH	HPGD
31	Phenazine biosynthesis-like domain-containing protein 2 *(C0H855)*	2.33	11	< 0.001	PBLD2	PBLD
32	Metallothionein B (MT-B) *(MTB)*	2.33	2	0.004	mtb	
33	Serpin H1 *(B5X1Q5)*	3.07	4	0.004	SERPH	SERPINH1
34	Ferritin *(C0H793)*	3.16	8	< 0.001	FRIM	FTL
35	Digestive cysteine proteinase 2 (B5X4D9)	3.63	7	< 0.001	CYSP2	
36	Canopy homolog 2 *(B5XA82)*	4.41	5	< 0.001	CNPY2	CNPY2
37	Erythrocyte band 7 integral membrane protein *(B5X1V0)*	5.01	6	0.005	STOM	STOM

Reported proteins with p-values < 0.05 after adjustment for multiple testing using the Benjamini Hochberg correction

Table 2.

Biological pathway analysis of regulated proteins using IPA software revealed 43 significant canonical pathways. The significance of the association between the data set and the pathway was determined based on the *p*-value, which determines the probability that the association between the data set and the pathway is explained only by chance, and on the ratio value, representing the number of proteins from the data found in each pathway over the total number of proteins in that pathway. The top five significant canonical pathways included *“EIF2 Signaling”* (*p*-value = 6.3 × 10^− 30^; ratio = 0.17), *“Protein Ubiquitination Pathway”* (*p*-value = 1.6 × 10^− 19^; ratio = 0.10), *“Regulation of eIF4 and p70S6K Signaling”* (*p*-value = 4.9 × 10^− 10^; ratio = 0.09), *“mTOR Signaling”* (*p*-value = 1.3 × 10^− 8^; ratio = 0.07) and *“Mitochondrial Dysfunction”* (*p*-value = 4.0 × 10^− 6^; ratio = 0.06), while those with the higher ratio included *“Spliceosomal Cycle”* (*p*-value = 2.0 × 10^− 2^; ratio = 0.5), *“Fatty acid β-oxidation”* (*p*-value = 1.5 × 10^− 3^; ratio = 0.33), *“Pentose Phosphate Pathway”* (*p*-value = 2.8 × 10^− 4^; ratio = 0.18), *“β-alanine degradation”* (*p*-value = 4.5 × 10^− 3^; ratio = 0.20), *“Superoxide radicals degradation”* (*p*-value = 4.5 × 10^− 3^; ratio = 0.20), *“Endoplasmic Reticulum Stress Pathway”* (p-value = 5.6 × 10^− 5^; ratio = 0.19) and *“Valine Degradation”* (*p*-value = 2.7 × 10^− 5^; ratio = 0.14). The activity pattern of the canonical pathway can be predicted by the activation z-score, a statistical measure of the match between expected relationship direction and observed expression [32]. Predicted activity was found to be increased for *“NRF2-mediated Oxidative Stress Response”* (*p*-value = 1.7 × 10^− 5^; ratio = 0.06; z-score = 0.45). The full list of canonical pathways as determined by temperature-regulated proteins is shown in Additional file 4.

Toxicity pathways are canonical pathways that are significantly associated with toxicity lists. These are functional groupings based on critical biological processes and key toxicological responses, and describe adaptive, defensive, or reparative responses to xenobiotic insults. The significance of the association is also defined by a *p*-value and a ratio value. A total of 15 significant toxicity pathways were mined from the temperature-regulated proteins. The five most significant pathways also showed the highest ratios and included *“Fatty acid metabolism”* (−log *p*-value = 1.6 × 10^− 25^; ratio = 0.09), *“Increases Transmembrane Potential of Mitochondria and Mitochondrial Membrane”* (*p*-value = 4.0 × 10^− 21^; ratio = 0.14), *“Mitochondrial Dysfunction”* (p-value = 1.3 × 10^− 19^; ratio = 0.06), *“Glutathione Depletion-CYP Induction and Reactive Metabolites”* (*p*-value = 2.5 × 10^− 18^; ratio = 0.33) and *“Oxidative Stress”* (−log *p*-value = 5.0 × 10^− 16^; ratio = 0.11). The components of the top five toxicity pathways are shown in Additional file 5.

**Fig. 3:**
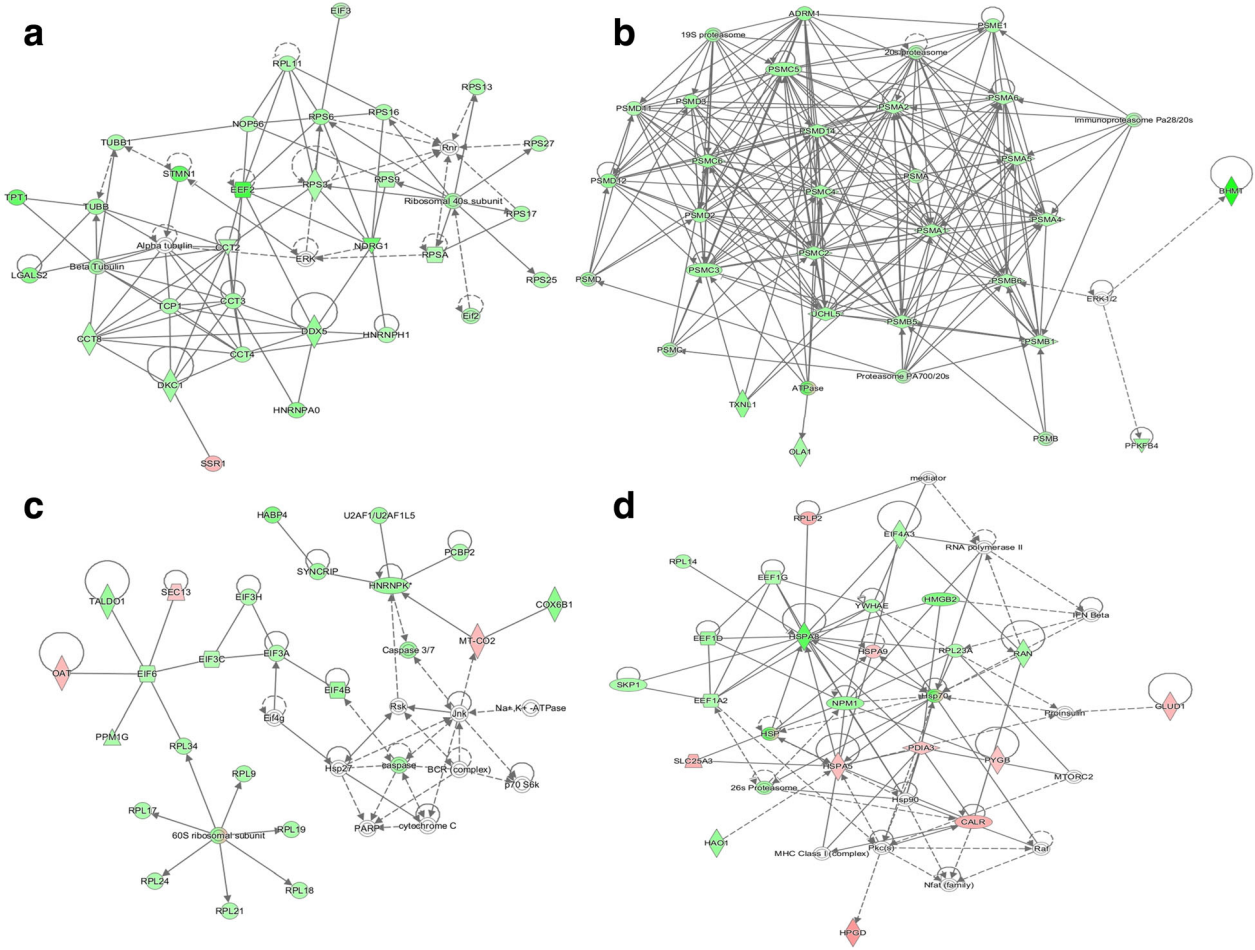
Top four-scoring biological networks for the significantly elevated temperature (21 °C) regulated proteins in liver of Atlantic salmon using IPA analysis. **a** RNA Post-Transcriptional Modification, Cellular Assembly and Organization, (**b**) Cell-To-Cell Signaling and Interaction”, “Cancer, Cell Death and Survival, Organismal Injury and Abnormalities, (**c**) Protein Synthesis, Gene Expression, Developmental Disorder, and (**d**) Protein Trafficking, Molecular Transport, Cellular Compromise. Nodes are colored according to increased (red) or decreased (green) abundance at elevated temperature. A line indicates that two proteins have shown binding, a line terminating in an arrow means that one protein acts on the other protein. Networks illustrate the effect of chronically elevated temperature on protein synthesis and degradation via down-regulation of ribosomal subunits (RPS proteins in network A and RPL proteins in network C), translation initiation (EIF proteins in network C) and elongation factors (EEF proteins in networks A and D), chaperoning-containing TCP1 complex (TCP1 and CCT proteins in network A) and proteasome subunits (PSMC and PSMD proteins in network B). See Additional file 3 for full name of node abbreviations

IPA software identified 15 top networks and ranked them by a score that considers the number of focus proteins and the size of the network to approximate the relevance of the network to the original list of focus proteins [32]. Networks with scores ≥2 have at least a 99% confidence level of not being generated by random chance alone. Top five networks revealed links with *“RNA Post-Transcriptional Modification, Cellular Assembly and Organization”* (score 52), “*Cell-To-Cell Signaling and Interaction”, “Cancer, Cell Death and Survival, Organismal Injury and Abnormalities”* (score 44), *“Protein Synthesis, Gene Expression, Developmental Disorder”* (score 39), *“Protein Trafficking, Molecular Transport, Cellular Compromise”* (score 37), and *“Lipid Metabolism, Small Molecule Biochemistry, Molecular Transport”* (score 35). The components of the top four networks are shown in Fig. 3.

## Discussion

We have used shotgun proteomics to characterize changes in the liver proteome of Atlantic salmon following exposure to chronically elevated temperature (21 **°**C). To our knowledge, this is the first study to provide proteome-level evidence of such response in Atlantic salmon. The metabolic role and plasticity of liver tissue in the thermal adaptive process was reflected in the clear separation between temperature treatments by MDS and in the high number of proteins that were differentially regulated at 21 **°**C. The general pattern of down-regulation is in line with the liver transcriptome response of similar size Atlantic salmon upon chronically elevated temperature [13]. The molecular dynamics of adaptation to thermal stress are dependent on tissue type examined, type of stress, and thermal exposure regime [11]. Specifically in liver tissue, metabolic down-regulation and small magnitude response of stress indicators appears to be associated with chronic exposure to elevated temperature [13, 33], while an up-regulated response and larger fold changes are elicited in response to more acute thermal stresses [15, 16].

Physiological adjustments to maintain tissue homeostasis were paralleled by constraints in performance. Metabolic rate increases exponentially as temperature increases and, at any given temperature, the difference between feed intake and metabolic rate will determine the energy available for tissue development and fish growth [10]. Given the similar feed intake between temperature treatments, the lower biometric indices (HSI, k) in the 21 **°**C fish suggested that the energy deficit caused by the increased energy demand was not met by feed intake. The proteomic data described here correlate with the biometric data and provide new insights into the main biological processes involved in the liver adaptive response of Atlantic salmon to chronically elevated temperature. We linked the pathways and networks generated by IPA analysis with expression patterns of protein groups and greatest fold changes to identify prevailing processes affected by elevated temperature and describe them in the following four sections.

### Protein synthesis and degradation

Protein synthesis is a major energy consuming process that can account for up to 42% of total energy expenditure in fish [34]. Of all tissues, the liver tissue has one of the highest rates of protein synthesis [34], and so is considered a highly sensitive physiological indicator of the impact of elevated temperature [35, 36]. Suppression of protein synthesis observed in the current study is consistent with previous liver transcriptional data in Atlantic salmon [13], and indicates a possible compensatory response to the energy deficit induced under chronically elevated sub-lethal temperature. In both studies, two independent mechanisms: eukaryotic initiation factor 2 (EIF2) signaling and the mechanistic target of the rapamycin (mTOR) signaling pathway were implicated. These regulatory mechanisms respond to energy status and stress within the cell via the activity of serine/threonine kinases and phosphatases through phosphorylation/dephosphorylation of EIF2α [37] and downstream effectors such as eiF4 and EEF2 [38]. Accordingly, reduced expression levels of ribosomal subunits (25 proteins), translation initiation (eight proteins) and elongation factors (five proteins), including EIF2, eiF4 and EEF2 subunits, were accompanied by reduced serine/threonine phosphatase activity (PP1CC and PPM1G).

Global repression of the translational machinery was also reflected by the inhibition of pre-translational regulatory mechanisms, and by inhibition of cytoplasmic chaperones that assist in the folding and trafficking of nascent proteins. Down-regulation of heterogeneous nuclear ribonucleoproteins (HNRNPK, HNRNPH1, HNRNPAB, HNRNPA0, SYNCRIP, PCBP2), responsible for packing and stabilizing freshly transcribed pre-mRNA [39], mirrored a reduced demand for mRNA export out of the nucleus to translation active sites in the cytoplasm. Noteworthy was the down-regulation of chromatin regulators, including high mobility group proteins (HMGB2 and HMGB3), the intracellular hyaluran-binding protein (HABP4) and nucleophosmin (NMP1). Chromatin remodelling appears to be a critical process in compensating for elevated temperature effects [40], as also suggested in the liver transcriptome analysis of heat-stressed eurythermal annual killifish (*Austrofundulus limnaeus*) [41]. In cytoplasm, reduced chaperone demand was indicated by the 2.5-fold down-regulation of the heat shock cognate 71 kDa protein (HSPA8), which contributes to the overall cytoplasmic folding by binding approximately 20% of newly translated proteins [42]. The chaperonin-containing TCP1 complex (TCP1, CCT2, CCT3, CCT4, CCT8) was also down-regulated. While the TCP1 complex is involved in the folding of only ~ 1% of newly synthesized proteins [43], these mostly include microtubule proteins, which were also regulated as a consequence of thermal stress, and are discussed below in the context of cytoskeletal integrity.

An important finding of this study was the inhibitory effect of chronically elevated temperature on protein degradation via the ATP-dependent ubiquitin-proteasome pathway. Proteins involved in ubiquitin conjugation (UBE2V1, UBE2D2, UBQLN4), ubiquitin ligase activation (SKP, TCEB2) and de-ubiquitination (UCLH5, ADRM1), and in the subsequent proteasome degradation (five PSMCs, five PSMDs, five PSMAs and one PSME), were down-regulated. This was additionally paralleled by the down-regulation of cathepsins (CTSD, CATM) involved in lysosomal protein degradation pathway. Specifically, CTSD has been previously measured as an indicator of protein degradation in response to temperature stress in fish [20, 44]. Signs of suppressed protein degradation were however not detected in the liver transcriptome of similar size Atlantic salmon exposed to chronically elevated temperature, in that case at 19 **°**C [13]. This observation corroborates the impact that the extra energy deficit generated by an additional rise of 2 **°**C has on protein metabolism; under restricted feeding, such a difference in rainbow trout reduced the liver rates of protein synthesis and degradation by ~ 35–55% [45]. Reduced protein degradation is therefore a compensatory mechanism that follows protein synthesis inhibition in order to maintain growth and conserve energy under a temperature-induced energy deficit not compensated by feed intake. This concept is well established at temperatures within the upper critical range [46], however, it also seems to apply to the chronic exposure to temperatures considered to be within the range of optimal thermal tolerance, albeit at the upper limit.

### Energy and lipid metabolism

The liver is a central hub for the storage and conversion of high-energy substrates. Under optimal temperature and feeding conditions, fish use amino acids rather than glucose as preferential energy source, while the contribution of fatty acid oxidation to energy production is correlated to dietary lipid levels [47]. At elevated temperature, energy metabolism is remodeled to compensate for the consequences of increased metabolic rate [10, 13]. Consistent with the trend observed in the plasma metabolome of Atlantic salmon [8], our proteomic data suggested an increased dependence on amino acids rather than glucose and fatty acids for energy production upon chronically elevated temperature. This was reflected in the up-regulation of mitochondrial enzymes involved in the degradation of valine (HIBADH, HIBC), tryptophan (AFMID, GCDH) and leucine (MCCC1, MCCC2, IVD) towards the formation of citric acid (TCA) cycle intermediates, and supported by the increased expression of TCA enzymes such as glutamate dehydrogenase (GLUD1) and aspartate aminotransferase (GOT2). The apparent increased TCA flux from amino acid catabolism was paralleled by signs of reduced glucose availability, including up-regulation of glycogen catabolic enzymes (PYGB, MTAP), down-regulation of several proteins involved in the pentose phosphate pathway (PGD, RPE, TKT, TALDO1), and the down-regulation of pyruvate kinase (KPYK). Mitochondrial β-oxidation of fatty acids also showed signs of suppression (ACAD11, ECI2). This may be linked to the fact that, under unrestricted feeding, TCA dependence on fatty acid oxidation diminishes with increasing exposure time to elevated temperature [8].

Increasing temperature increases the fluidity of cell membrane, leading to alterations in lipid metabolism to maintain and stabilize fluidity [3, 48]. In this study, several proteins associated with fatty acid metabolism and lipid transport were down-regulated at elevated temperature and linked to mechanisms involved in modulating membrane fluidity. The peroxisomal acyl-coenzyme A oxidase 3 (ACOX3), the rate-limiting enzyme in the oxidative breakdown of methyl-branched fatty acids [49], showed the largest fold change in abundance (× 5.4). Since methyl-branched fatty acids have been suggested to enhance the fluidity of the membrane lipid bilayers over different environmental conditions [50], ACOX3 down-regulation may be part of a mechanism to increase membrane fluidity at elevated temperature. Membrane fluidity is stabilized by the incorporation of cholesterol into lipid bilayers, with increased cholesterol levels associated to increased temperatures [51]. An important mechanism in cholesterol homeostasis is reverse cholesterol transport (RCT), whereby cholesterol is transported in high-density lipoproteins (HDL) from peripheral tissues back to the liver to be further eliminated in the bile [52]. Elevated temperature appeared to inhibit RCT, as reflected by the parallel down-regulation of apolipoprotein A1 (APOA1), the major component of HDL, and lipase (LIPA), involved in HDL uptake and a well-known rate-limiting enzyme in RCT [52]. Subsequent signs of altered cholesterol metabolism included down-regulation of epididymal secretory protein E1 (NCP2), an intracellular cholesterol transporter that regulates cholesterol biliary secretion, and of 3-oxo-5-beta-steroid 4-dehydrogenase (AKR1D1), involved in cholesterol breakdown towards the synthesis of bile acids. These observations re-emphasize the importance of cholesterol in the acclimatization to elevated temperature [41], and further implicate suppression of RCT and cholesterol catabolism to increase peripheral retention under conditions of thermal stress.

### Cytoskeletal integrity

Stress-induced perturbation of transcription and translational mechanisms can block cell growth and proliferation via effects on cytoskeletal integrity [53]. Thus, the microtubule network of α- and β-tubulins forming the cell cytoskeleton is recognized to play a role in maintenance of cell homeostasis and execution of a variety of cell stress responses [54]. In line with a previous observation in the liver transcriptome of Antarctic fish [55], the liver proteome of Atlantic salmon reflected suppression of the microtubule dynamics in response to chronically elevated temperature. Down-regulation of tubulins (TUBB, TUBB1) was paralleled by down-regulation of the chaperonin-containing TCP1 complex (TCP1, CCT2, CCT3, CCT4, CCT8), which is particularly implicated in the folding and assembly of tubulins in an ATP-dependent manner [43]. Notably, microtubule-stabilizing proteins were also down-regulated, including tubulin-folding factor (TBCB) [56], translationally-controlled tumor protein (TPT1) [57], N-myc downstream regulated gene 1 (NDRG1) [58], and stathmin (STMN1) [59]. We highlight the larger than two-fold changes in TPT1 and NDRG1, which are established hallmarks and mediators of cell proliferation through microtubule stabilization. A novel outcome of the heat-induced cytoskeleton remodeling was the striking (4.4-fold) down-regulation of the canopy homologue 2 (CNPY2). CNPY2 is a transmembrane protein that regulates myosin regulatory light (MRLC) protein levels, a protein that links cytoskeleton to membrane proteins and stimulates cell growth [60]. Reduced cytoskeletal integrity at elevated temperature has been attributed to maintenance of a less dense subcellular structure resulting from alterations in cytosol solubility and viscosity [55], or to an increase in the pool of soluble tubulin as a result of oxidative stress-induced microtubule depolymerization [54]. In contrast to our results, acute heat stress induced up-regulation of genes encoding cytoskeleton components in gill tissue of Pacific salmon [61]. This difference between studies highlights the tissue- and exposure regime- specificity of the heat stress response across different protein groups and biological processes [11].

### Oxidative stress and endoplasmic reticulum (ER) stress

Exposing ectotherms to elevated temperatures challenges the cellular redox balance and leads to increased production of reactive oxygen species (ROS) [62]. Mitochondria are well-known as major sites of ROS production and consumption and are implicated as the main source of thermally-induced oxidative stress [63]. ROS imbalance occurs due to the uncoupling of mitochondrial respiration; a proton gradient generated by complex I, III and IV, but not coupled with consumption by complex V, will leak, consequently increasing the mitochondrial membrane potential and ROS formation [64, 65]. This mechanism, previously proposed as a cause of ROS formation in polar and temperate fish upon long-term warm acclimation [66], is consistent with our findings here in Atlantic salmon, with imbalances in the electron transport complex IV (COXAI2, COX6B1, COX7A2L, MT-CO2) and complex V (ATPL5L) proteins and in other mitochondrial proteins (CAT, SOD2, CASP3, MSRB2) indicative of mitochondrial dysfunction and increased mitochondrial transmembrane potential. The ROS-induced cellular antioxidant response was predicted to be mediated via nuclear factor erythroid 2-related factor 2 (Nrf2) through the regulation of different mechanisms, which as previously described [67] included induction of catabolism of the ROS superoxide through mitochondrial superoxide dismutase (SOD2) and the peroxiredoxin system, and metal chelation by ferritin (FTL). The members of the peroxiredoxin family exhibited opposing patterns of regulation (e.g. reduced PRDX 1 and PRDX6 and increased PRDX3 and PRDX4) that are characteristic of other disorders related to oxidative stress [68] and attributed to the increased exposure to the superoxide derivative hydrogen peroxide [69, 70]. Specifically, the observed co-regulation of PRDX3 and PRDX4 has been proposed as an outcome of antioxidant response and oxidative damage in cancer [71]. The 3-fold up-regulation of FTL was similar to the increased transcript and protein abundance in *Channa* liver upon chronically elevated temperature [16]. Iron interacts with hydrogen peroxide via the Fenton reaction leading to the production of hydroxyl radical ROS, a very reactive initiator of lipid peroxidation [72]. As FTL maintains iron homeostasis, overexpression is proposed as an attempt to prevent Fenton type reactions and ROS accumulation [73]. The Nrf2-mediated antioxidant response was further supported by up-regulation of metallothionein B (mtb), a cysteine-rich metal binding protein involved in metal homeostasis and ROS scavenging [74]. Induction of the ROS scavenging system was also paralleled by the up-regulation of cytosolic epoxide hydrolases (EPHX1, EPHX2) and aldo-keto reductases (AKR7A2, AKR1B1), respectively involved in the detoxification of fatty acid epoxides and aldehydes [75, 76], and suggesting some of degree of lipid peroxidation in 21 **°**C livers, as previously detected in heat-stressed fish [77, 78]. A contribution of our study towards biomarker discovery was the significant and negative correlation between FTL expression and HSI across fish of both 15 **°**C and 21 **°**C groups (Figure 2). FTL is used as a reliable blood marker of liver disorders in human medicine [79], and it would be interesting to further explore the FTL expression levels in blood as a surrogate and less invasive measurement of liver condition and oxidative stress in fish.

Oxidative stress is a condition of imbalance between the formation of ROS and the biological system’s ability to detoxify the reactive intermediates, thus dysfunction of the antioxidant and detoxifying activity is also reflective of oxidative damage [63]. This concept is consistent with previous thermal stress studies in fish examining varying exposure regimes [11, 62] and was supported here by the down-regulation of catalase (CAT), glutathione transferases (GSTs: GSTT1 and GSTP1), and betaine-homocysteine S-methyltransferase 1 (BHMT) at elevated temperature. CAT is a mitochondrial and peroxisomal consumer of hydrogen peroxide, though down-regulation in fish has been attributed to its sensitivity to the fluctuation of superoxide radicals [80]. Glutathione plays a central role in the cellular defence against lipid peroxidation [81]. While GSTs catalyse the metabolism of lipid peroxides by conjugation with glutathione and NADPH [82], BHMT maintains steady levels of the glutathione precursor s-adenosyl-methionine (SAM) (i.e. SAM is the amino acid methionine bound to an ATP molecule) [83]. Co- regulation of GSTs and BHMT was thus indicative of glutathione depletion and reduced hepato-protection, as previously detected and associated to increased lipid peroxidation in liver of fish exposed to thermal stress [15, 77, 78]. These results collectively target the methionine cycle as a possible route to mitigate the impaired enzymatic hepato-protection associated to thermal stress. Enhanced protection was recently suggested in seabream fed a winter-specific diet supplemented with methionine [84]. A comparison of our proteomic data with the liver transcriptome of similar size Atlantic salmon upon chronic exposure to 19 °C [13] implicates variation in the oxidative stress response that attributes to temperature-specific effects and the consequent ROS production [11, 63]. This highlights the risk of drawing general conclusions and also the importance of other experimental factors such as dietary formulation (e.g. antioxidant supplementation) and methodology used (e.g. proteomics vs. transcriptomics, pooling vs. individual-based analysis) in identifying thermal stressors across studies.

There is accumulating evidence for an intrinsic link between cellular oxidative stress and ER stress [85]. One proposed mechanism for this interrelation is that mitochondrial ROS formation promotes the calcium release from the ER, leading to the accumulation of unfolded proteins in the ER lumen and further contributing to ROS formation due to the excessive calcium influx into the mitochondria [86]. The ER unfolded protein response (UPR) is subsequently activated and mediated through the induction of molecular chaperones to restore proteostasis and avoid apoptosis. At elevated temperature, activation of the UPR response was indicated by the up-regulation of chaperones and other proteins required for protein folding and stabilization of pre- and un-folded proteins, specifically calreticulin (CALR), disulfide isomerases (PDIA3, PDIA6), 78 kDa glucose-regulated protein (HSPA5/Bip or GRP78) and ER glycosyltransferases (UGT2A1, RPN1). Our data confirm previous detections of ER stress and UPR in liver of thermal-stressed fish [15, 16, 33, 84] and demonstrate its specific importance in the adaptive response to chronically elevated temperature. Another indicator of the crosstalk between cellular oxidative stress and ER stress was the 3-fold up-regulation of the collagen-specific chaperone Serpin H1 (SERPINH1 or HSP47) which is also induced by stress induced-lipid peroxidation [87, 88]. In line with our study, increased transcript levels of SERPINH1 were also found in gill tissue of Pacific salmon under different heat exposure regimes [61, 89, 90].

## Conclusions

This study increases our understanding of the molecular mechanisms occurring in the liver of pre-harvest Atlantic salmon that are important for coping with chronically elevated temperature, which is of increasing importance in temperate production areas such as Tasmania. Suppression of protein synthesis and degradation pathways appears to be the main energy saving mechanism for the increased metabolic demand, which is also reflected in an increased dependence of amino acid catabolism towards energy production. Other chronic heat-stress related mechanisms included the reverse transport and catabolism of cholesterol, cytoskeletal dynamics, the Nrf2-mediated antioxidant response and the endoplasmic reticulum UPR. Many of the proteins regulated here are common to the thermal stress response across fish species and tissues. These included protein groups (TCP1 complex proteins, high mobility group proteins, disulfide isomerases, peroxiredoxins and glutathione transferases) and individual proteins (CTSD, SOD2, FTL, BHMT, HSPA5, CALR and SERPINH1). Other stressors, particularly several cytoskeletal-related proteins (STMN1, TPT1, NDRG1 and CNPY2) were for first time here reported in response to thermal stress. Finally, opportunities for new research towards the development of salmon feed formulations specific to the summer period are raised here. Further understanding of the potential for methionine supplementation to improve the liver detoxifying capacity, or the evaluation of ingredients and additives to compensate the energy deficit and to spare amino acid degradation towards energy production are warranted.

## Additional files

Additional file 1: MaxQuant output files of the complete peptide-level mass spectrometry. A total of nine biological replicates (fish) per temperature treatment were analysed. (XLSX 5578 kb)

Additional file 2: MaxQuant output files of the complete protein-level mass spectrometry. A total of nine biological replicates (fish) per temperature treatment were analysed. (XLSX 2354 kb)

Additional file 3: Total number of quantifiable proteins. Proteins identified on the basis of two or more unique matching peptide sequences and presence in at least six of the nine biological replicates in either treatment group (XLSX 160 kb)

Additional file 4: Full list of canonical pathways as determined by temperature-regulated proteins and predicted by IPA analysis. The significance of the association between the data set and the pathway is based on the *p*-value, which determines the probability that the association between the data set genes and the pathway is explained only by chance, and on the ratio value, representing the number of genes from the data found in each pathway over the total number of genes in that pathway. (XLSX 56 kb)

Additional file 5: Components of the top five toxicity pathways as determined by temperature-regulated proteins and predicted by IPA analysis. Exp fold change and Exp p-value correspond with the fold change value and the adjusted p-value (using Benjamini Hochberg correction), respectively, reported in Additional file 3. (XLSX 52 kb)
